# Gen Z Tourism Employees’ Adaptive Performance During a Major Cultural Shift: The Impact of Leadership and Employee Voice Behavior

**DOI:** 10.3390/bs15020171

**Published:** 2025-02-05

**Authors:** Kleanthis K. Katsaros

**Affiliations:** Department of Food Science & Technology, School of Agricultural Sciences, University of Patras, G. Seferi 2, 30100 Agrinio, Greece; klekatsaros@upatras.gr

**Keywords:** adaptive leadership, adaptive performance, Gen Z employee, inclusive leadership, transformational leadership, voice behavior

## Abstract

Based on social exchange theory and the norm of reciprocity, the current study proposes a mediation model to assess the role of employee voice behavior (promotive and prohibitive) on the relationship between leadership (i.e., transformational, inclusive, and adaptive) and Gen Z employees’ adaptive performance (AP). Research data were obtained from 195 Gen Z employees and their supervisors from a group of luxury hotels located in Greece that had experienced a major cultural shift. The research model was examined using the structural equation modeling technique (SEM) with maximum likelihood estimation using the analysis of moment structures program (AMOS version 24). The research findings indicate that (a) all three leadership approaches positively influence Gen Z employees’ AP, (b) promotive voice behavior mediates the relationship between all leadership approaches and Gen Z employees’ AP, and (c) prohibitive voice behavior mediates the relationship between inclusive leadership and Gen Z employees’ AP. The results suggest that by implementing human-centered practices and procedures to positively influence Gen Z employees’ voice behavior, tourism leaders/managers can increase their AP.

## 1. Introduction

Today, organizations face difficulties in quickly adapting to the growing uncertainty, ambiguity, instability, volatility, and complexity in their environments (Katsaros, 2024). It is widely accepted that change management is above all an anthropocentric procedure that emphasizes the significance of people during change efforts (Schwarze & Taylor, 2017). That is, the adaptive challenges facing organizations these days require greater involvement and contribution from their employees who engage in the change initiatives (Carnevale et al., 2017). Thus, it is not surprising that developing employees’ adaptive performance (i.e., employees’ adaptability to the changes in the workplace; Park & Park, 2019) has emerged as a primarily significant issue in both theory and practice. As a result, organizations should try to adopt democratic, participatory, and people-oriented policies and procedures that may increase employees’ adaptive performance (Vakola et al., 2021).

It is well documented that leadership and organizational change are two closely related terms (e.g., Peng et al., 2021). In the end, leadership refers to the ability to implement change by articulating and communicating a vision to others (Boyles, 2024). Given that capable leadership is essential for effective change implementation (e.g., Vito et al., 2023), numerous studies have explored the impact of leadership on employee adaptive performance. Some of them have expanded upon the role of inclusive leadership, given that leaders should encourage organizational members to change their behaviors by supporting them to find out their role during change (Katsaros, 2024). Other studies have explored the role of transformational leadership as it may facilitate employees in seeking new solutions to problems, adapting to change, exceeding performance expectations, and demonstrating heightened commitment in tricky situations (Park & Park, 2019). Quite from a different perspective, some of them have explored the role of adaptive leadership as being a more follower-centric approach that facilitates employees in handling difficult situations and overcoming change-related obstacles (Naseer et al., 2023).

However, examining these leadership approaches in isolation can be quite tricky. That is, the leadership style should adapt and respond to various situations. According to the theory of dynamic leadership (Mostovicz, 2009), a leader should employ a flexible leadership style to accommodate the specific needs of the organization. There is a dearth of empirical research regarding how all of these leadership approaches may concurrently influence employee adaptive performance and, consequently, the factors that may explain these important relationships. The examination of the way these leadership approaches might simultaneously influence employee adaptive performance is the key and innovative area of the current study. Exploring how transformational, inclusive, and adaptive leadership styles interact can provide a more holistic understanding of leadership’s impact on performance in dynamic environments. By drawing on social exchange theory (i.e., individuals exhibit a tendency to reciprocate positive actions with equally valued behaviors; Blau, 1964) and the norm of reciprocity (i.e., individuals experience an intrinsic obligation to reciprocate positive organizational treatment by displaying positive workplace attitudes; Gouldner, 1960), the paper tries to bridge this theoretical gap by proposing a mediation model to assess the relationship between leadership (i.e., transformational, inclusive, and adaptive) and employee adaptive performance through employee voice behavior (promotive and prohibitive). That is, on the one hand, positive leadership approaches such as ethical, authentic, paternalistic, transformational, inclusive, and/or servant may stimulate employee voice behavior (e.g., Weiss et al., 2017; Yan & Xiao, 2016), while on the other hand, employee voice behavior entails a behavior that argues the status quo and is change oriented, yet with an intention to be positive and beneficial (e.g., reflects employees’ eagerness to participate in discussions about changes and to offer constructive feedback; Li et al., 2020).

The current study offers three significant contributions to the existing literature. First, it responds to the call for increased empirical evidence on employee adaptive performance (Park & Park, 2021). So far, several researchers have evaluated employee adaptive performance, but rather few studies have examined Gen Z employees’ adaptive performance and its possible outcomes (e.g., Katsaros, 2024). This is rather disturbing in view of the fact that Gen Z is now the most populous generation on Earth (Jeremiah, 2020), and it is anticipated that it will constitute 27% of the global workforce by the year 2025 (OECD, 2022). This new direction will benefit both researchers and practitioners. Second, although researchers have extensively investigated employees’ reactions during change (e.g., Oreg et al., 2023), unexpectedly, they have not fully expanded on how these positive leadership approaches may interchangeably promote participative and constructive behaviors during change efforts (e.g., Katsaros, 2022). Failure to do so gives rise to rather poor change management practices. Third, the research contributes to the theory of change in tourism (i.e., understanding the reasons and processes behind change in this industry, how targeted interventions can achieve desired outcomes, and how to effectively manage both employees and organizations; Twining-Ward et al., 2018) by pinpointing the significance of employee voice behavior (promotive and prohibitive) during change initiatives.

## 2. Literature Review and Hypotheses Development

Transformational leadership (hereafter, TL) refers to a leadership approach that refers to inspiring and motivating employees to reach their highest potential and drive positive change within an organization (Jones, 2018). Bass and Avolio (1995) were the first to identify the four components of TL, namely, idealized influence, inspirational motivation, intellectual stimulation, and individualized consideration. Through such behaviors, transformational leaders may exert a positive influence on employees’ attitudinal and behavioral responses, thereby increasing their creativity, self-motivation, job satisfaction, and performance (e.g., Puni et al., 2018). As a result of this powerful function of TL, several researchers have applied this approach to organizational change (e.g., Abrell-Vogel & Rowold, 2014). These authors have examined the positive effects of TL on employees’ change reactions, which comprise change commitment, openness to change, readiness for change, and change support, as well as reduced resistance and cynicism (e.g., Peng et al., 2021). Within this context, Islam et al. (2021) suggested that a transformational leader typically serves as a catalyst for change by increasing employees’ awareness of collective benefits and empowering them to attain extraordinary objectives.

Inclusive leadership (hereafter, IL) refers to building relationships that can reach goals through mutual benefit, indicating that leadership is more about collaborating with people rather than simply acting on their behalf (Katsaros, 2022). This approach departs from the habitual and long-standing focus on the qualities/abilities/behaviors/characteristics of a leader by focusing on the followers’ perspectives and aspirations. Moreover, it is oriented towards the followers’ involvement (rather than their manipulation), respect, recognition, and accountability, which are vital to the successful implementation of positive leadership. Also, it has a human-centered character and approach, exactly as change management should have nowadays, consistent with the international literature (e.g., Regine, 2020). It is important to note that IL differs from TL. A transformational leader provides encouragement and personal support while challenging existing assumptions and generating new ideas. By contrast, an inclusive leader emphasizes externalization and accessibility to involve individuals in a dynamic process that values mixed viewpoints (Mitchell et al., 2015). According to Shore and Chung (2022), inclusive leaders listen carefully to employees’ opinions, embrace their failures, and they offer support and guidance when needed. Quite in the same way, Qi et al. (2019) emphasized that IL values employees’ contributions and prioritizes their well-being and psychological needs, which can foster their ingenuity, creativity, and innovation. Numerous research studies suggest that IL is regarded as imperative during change. For example, Qurrahtulain et al. (2022), by relying on the affective events theory, showed that vigor at work mediates the relationship between IL and adaptive performance, and Katsaros (2024) concluded that leaders and managers, to help Gen Z employees adapt to change, should focus on making them feel engaged and satisfied by using inclusive practices.

Adaptive leadership (hereafter, AL) enables individuals and organizations to adjust to changing environments and effectively tackle arising challenges (Heifetz et al., 2009). This leadership approach can promote understanding among all hierarchical levels and foster a culture of experimentation, innovation, and continuous learning. It is an anthropocentric approach that assists employees ineffectively confronting difficult situations and overcoming change-related obstacles (Naseer et al., 2023). The basic assumption of this approach is that today there are two kinds of problems, namely, technical and adaptive. Technical problems are mechanistic; there are already tested and acceptable answers for them can be obtained from corresponding past experiences. By contrast, adaptive problems are vague and unclear, and there are no predefined rules or procedures for handling them (e.g., the outbreak of the COVID-19 pandemic). In such cases, the expertise of an adaptive leader is useful in both defining the problem and mobilizing organizational members to find solutions (Mulder, 2023). The application of adaptive leadership to organizational change efforts can significantly enhance the human dimension of change by emphasizing the behaviors and competencies necessary for leaders to motivate followers to flourish during periods of transformation (Arthur-Mensah & Zimmerman, 2017). Further, AL notes the significance of leadership as a learning process in which leaders and employees collaboratively experiment with new ideas to develop innovative solutions. As a result, employees experience a sense of commitment in the change process, and they are better equipped to navigate the uncertainty, ambiguity, and volatility inherent in organizational change (Northouse, 2016).

### 2.1. Leadership and Employee Adaptive Performance

Recent research strongly proposes that employee adaptive performance (hereafter, AP) is imperative for change success (e.g., Katsaros, 2024; Vakola et al., 2021). AP refers to how well employees can deal with changes at work (Park & Park, 2019). It represents a behavior rather than an individual tendency, and it can be exhibited both in anticipation of and in response to a change initiative (Jundt et al., 2015). At the individual level, it can lead to a range of positive outcomes, such as increased job satisfaction (Marques-Quinteiro et al., 2019), work engagement (Kaya & Karatepe, 2020), and performance capability (Shoss et al., 2012). It can also result in significant organizational outcomes, such as effective management of change and enhanced organizational knowledge (Camps et al., 2016; Dorsey et al., 2017). According to Jundt and Shoss (2023), AP research has mainly examined skill-based adaptation; however, AP may also refer to adapting one’s interpersonal or emotional responses to change. Because it has been studied in various contexts and using different variables, formulating a universal definition or approach to AP is quite challenging (Park & Park, 2019). The paper examines AP as reflecting an individual’s efforts (i.e., cognitive, emotional, and behavioral) to adapt during organizational changes.

Studies on group characteristics have noted that leadership represents a rather significant predictor of individual AP (Chiaburu et al., 2013; Park & Park, 2019). In more detail, TL at the team level may contribute to the emergence of AP by empowering employees to seek innovative solutions to problems, adapt to change, exceed performance expectations, and exhibit commitment in complex situations (Park & Park, 2019). For example, Curado and Santos (2022), in examining health-care professionals, found that job satisfaction fully mediates the relation between TL and AP, and Buttigieg et al. (2023), in studying 432 participants at three hospitals in Malta, found positive relationships among TL, leadership agility, work engagement, and AP. Quite similarly, IL values employees’ contributions, cares about their psychological well-being, and thus, it may increase creativity, innovation, and change. In the same vein, Fatima et al. (2021) showed that IL is positively related to being driven at work and that employees’ readiness for change mediates this relationship, and Katsaros (2024), in examining 305 Gen Z employees in the Greek telecommunication industry and their supervisors, concluded that happiness in the workplace partially mediates the relationship between IL and employee AP. Finally, AL, as a leadership model that notes the capacity to navigate complex and changing environments, focuses on the importance of leaders being flexible and responsive to challenges while empowering their team members to tackle difficult problems (Uhl-Bien & Arena, 2018). For example, Naseer et al. (2023) found that high AL and high-performance work practices establish the conditions for cultivating an individual’s affective commitment to change via their readiness to change, and Fausett et al. (2024) noted the importance of AL in health care to foster teamwork, improve patient care, and create positive work environments.

Overall, social exchange theory strongly indicates that when employees receive positive behavior from someone, they will likely respond with something of similar significance (Blau, 1964), and the norm of reciprocity suggests that employees feel an inherent obligation to repay the organization for positive treatment they have received by demonstrating positive attitudes in the workplace (Gouldner, 1960). Thus, if employees receive positive behaviors, respect, sincerity, fairness, and empathy from their leaders during change initiatives, they will feel compelled to reciprocate with positive change-related behaviors (Garba et al., 2018). According to the above rationale and the aforementioned research findings, we hypothesize:

**Hypothesis** **1.** *Transformational leadership is positively associated with employee adaptive performance.*

**Hypothesis** **2.** *Inclusive leadership is positively associated with employee adaptive performance.*

**Hypothesis** **3.** *Adaptive leadership is positively associated with employee adaptive performance.*

### 2.2. The Mediating Role of Employee Voice Behavior

Voice behavior is a proactive behavior that involves expressing a positive challenge aimed at improvement (rather than just criticism), and it includes making innovative suggestions for enhancements and proposing changes to established procedures (Van Dyne & LePine, 1998). It represents an active behavior that depicts employees’ willingness to contribute to discussing organizational changes and proposing positive alternatives. It can significantly facilitate employee adaptive performance through several mechanisms, such as positive communication (Kim & Lim, 2020), creativity and innovation (Carnevale et al., 2017), trust and engagement (Holland et al., 2017), employee empowerment (Yoo, 2017), and/or positive organizational culture (e.g., an environment that fosters psychological safety, encouraging individuals to take risks and adapt without fear of negative consequences; Joseph & Shetty, 2022).

Voice behavior can be classified as promotive or prohibitive. Promotive voice behavior refers to employees’ proactive communication of ideas, suggestions, and/or concerns aimed at improving organizational practices or addressing issues within the workplace (Liang et al., 2012), whereas prohibitive voice behavior involves employees raising doubts, objections, or warnings about potential issues or unethical practices within an organization, along with suggesting relevant solutions (Liang et al., 2012). Unlike promotive voice behavior, which focuses on proposing developments or innovations, prohibitive voice behavior is mainly concerned with preventing negative outcomes, such as noting risks, ethical violations, or practices that could harm the organization or its members. Overall, employees are expected to be accountable for innovation, improvements, and change when organizations offer resources to sustain new proposals and care about employees’ voices and views (Chiaburu et al., 2013). Thus, we hypothesize:

**Hypothesis** **4.** *Promotive voice behavior is positively associated with employee adaptive performance.*

**Hypothesis** **5.** *Prohibitive voice behavior is positively associated with employee adaptive performance.*

As a consequence of its structural relationship with antecedents (e.g., individual characteristics, contextual factors, social exchange, and psychological factors) and consequences (e.g., individual and organizational performance), voice behavior has gained significant attention from HRM researchers and practitioners (Chou & Barron, 2016). On the one hand, the empirical evidence indicates that factors influencing voice behavior may include positive leadership approaches, such as ethical leadership (Chen & Hou, 2016), authentic leadership (Liang, 2017), paternalistic leadership (Zhang et al., 2015), transformational leadership (Duan et al., 2016), inclusive leadership (Qi & Liu, 2017), servant leadership (Yan & Xiao, 2016), and paradoxical leadership (Li et al., 2020). On the other hand, employee voice behavior refers to a positive and helpful change-oriented communication intended to alter the status quo of the organization and improve the current situation (Chen & Hou, 2016), and there is empirical evidence that employee voice behavior (promotive and prohibitive) may provoke positive individual and organizational change-related outcomes (e.g., Maynes et al., 2024; Naqvi, 2020; Potnuru et al., 2023). Further, quite similarly, social exchange theory (Blau, 1964) and the norm of reciprocity (Gouldner, 1960) suggest that employees will feel a natural obligation to reciprocate the positive and human-centered treatment they have received from their organization’s leadership. According to the above rationale and the research findings, we hypothesize:

**Hypothesis** **6.** *Promotive voice behavior mediates the positive relationship between leadership (i.e., transformational, inclusive, and adaptive) and employee adaptive performance.*

**Hypothesis** **7.** *Prohibitive voice behavior mediates the positive relationship between leadership (i.e., transformational, inclusive, and adaptive) and employee adaptive performance.*

**Figure 1:**
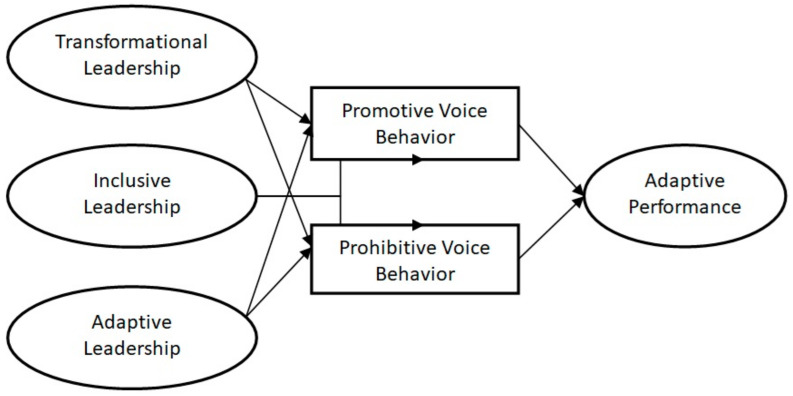
Research model (created by the author).

Thus, we have the following research model (Figure 1).

## 3. Research Background

Greece is a very popular tourist destination, ranking 9th in the world with 27.8 million international tourist arrivals in 2022 (WTO, 2024). Consequently, the tourism industry plays a vital role in the Greek economy. In 2022, the total contribution of travel and tourism to Greece’s GDP was approximately 38 billion Euros, accounting for nearly 17.5% of the GDP—only 7.4% lower than in 2019, the year before the COVID-19 pandemic. Moreover, it considerably impacts employment, providing around 800,000 jobs, or nearly 19.3% of total employment in Greece (Statista Research Department, 2024). Currently, the tourism sector is facing major challenges due to (a) the rise of the home-sharing economy, which has intensified price competition in the hospitality industry; (b) the “green transition” aimed at addressing the climate crisis; (c) digital transformation; (d) new social distancing standards as a legacy of the pandemic; and (e) efforts to promote accessibility and inclusiveness (Alpha Bank Economic Research, 2022). Consequently, the Greek tourism industry is undergoing a significant strategic transformation, prioritizing the extension of the tourist season, the attraction of high-value tourist segments, the increase in average daily expenditure, and the exploration of emerging tourist markets. In this context, Greece is committed to making substantial investments in the tourism sector over the forthcoming years, with the objective of evolving its traditional tourist offerings into a diverse range of highervalue and more specialized products (Enterprise Greece, 2024).

Gen Z, defined as those born between 1995 and 2012, comprises approximately 2 billion people globally and is expected to represent 27% of the workforce by 2025 (McAllister, 2024). This generation is continuously entering the tourism industry and is poised to become the largest cohort (Goh & Okumus, 2020). Compared to previous generations, Gen Z displays a distinctive set of workplace values (Sakdiyakorn et al., 2021). They seek to feel valued, included, respected, supported, and motivated (Mathew, 2023). Additionally, they place a high premium on workplace happiness, actively looking for positive workplaces that endorse work–life balance, highlight purpose-driven work, value diversity, support personal contributions, and prioritize employees’ psychological well-being (Linh, 2024). These traits shape their expectations as employees, informing how organizations should engage with them.

## 4. Methods

### 4.1. Procedure and Participants

Participants in this study were Gen Z employees from a group of luxury hotels in Greece. The group had experienced a significant cultural transformation driven by the new, competent leadership. Specifically, there was a transition from a hierarchical culture, characterized by top–down decisionmaking, to a more adhocratic culture, emphasizing flexibility, innovation, and a people-centered approach (Cameron & Quinn, 1999). This cultural shift emphasized decentralized decisionmaking, honest collaboration, risktaking, and experimentation, and at the same time, it focused on meeting the needs of both employees and customers. As a result, this group of luxury hotels would be better equipped to respond to rapidly changing environments and evolving market demands (Cameron & Quinn, 1999). According to the leaders of the group, the most challenging aspect of this cultural shift was the employees’ difficulty in handling the emergent uncertainty, which led to increased levels of stress and anxiety, particularly during the initial stages of the change process. We used total population sampling (i.e., a purposive sampling technique that involves examining the entire population) and, in close cooperation with the organization, all Gen Z employees were invited to voluntarily participate in the present research. On the whole, 195 employees (response rate of 78.00%) and 22 supervisors (response rate of 66.00%) participated in the study. Most of the employees were women (61.54%), they had at least a bachelor’s degree (51.28%), and they had total working experience of 5–10 years (89.74%). Quite differently, most of the supervisors were men (68.18%), they had at least a master’s degree (81.81%), and they had working experience of 5–10 years (59.09%). Finally, the majority of the sample could be placed into three different categories: guest services (55%), support staff (20%), and administration (15%).

We adopted a procedural design as outlined by Podsakoff et al. (2003). In the questionnaire’s instructions, we specified that the study results would be used solely for this academic research and that all information would be kept strictly confidential. We then gathered data through a phased approach, collecting data in three waves approximately four months apart, recognizing that cultural changes require time to significantly alter deeply ingrained behaviors and norms (Heskett, 2021). In the first phase, employees assessed the leadership approach (i.e., transformational, inclusive, and adaptive). In the second phase, supervisors evaluated their subordinates’ voice behavior (i.e., promotive and prohibitive). Finally, supervisors assessed the adaptive performance of the employees. Utilizing different sources of data (i.e., supervisors and employees) helped to mitigate the risk of common method variance (Podsakoff et al., 2003). The questionnaires included employee identification codes to allow for matching and grouping of data from supervisors and employees for further analysis. Participation in the research was voluntary. One week before the study commenced, the research team informed participants about the study’s objectives during an e-presentation, after which they received a survey e-package containing instructions and guidelines. This e-package involved a cover letter and detailed completion instructions. The research team provided support at all three stages (both online and onsite) to ensure the study’s quality.

### 4.2. Measures

All questionnaire items were measured on a five-point Likert-type scale ranging from ‘strongly disagree’ (1) to ‘strongly agree’ (5). Transformational leadership (employee rated) was measured with the 7-item scale developed by Carless et al. (2000). An example item is “My leader communicates a clear and positive vision of the future.” Inclusive leadership (employee rated) was measured with the 9-item scale developed by Carmeli et al. (2010). An example item is “The leader is ready to listen to my requests.” Adaptive leadership (employee rated) was measured with the 15-item scale developed by Northouse (2016). An example item is “When people feel uncertain about organizational change, they trust that this leader will help them work through the difficulties.” Promotive voice behavior (supervisor rated) was measured with the 5-item scale developed by Liang et al. (2012). An example item is: “This employee proactively develops and makes suggestions for issues that may influence the unit.” Prohibitive voice behavior (supervisor rated) was measured with the 5-item scale developed by Liang et al. (2012). An example item is: “This employee advises other colleagues against undesirable behaviors that would hamper job performance.” Adaptive performance (supervisor rated) was measured with the 3-item scale developed by Griffin et al. (2007). An example item is “This employee has adapted well to the changes in his/her core tasks.”.

## 5. Results

Table 1 presents the descriptive statistics, correlations, and reliabilities for the research variables. The mean values are 4.15 for TL (sd = 1.08), 4.22 for IL (sd = 1.11), and 3.88 for AL (sd = 0.77). Further, the mean values of promotive and prohibitive voice behavior are 4.44 (sd = 1.01) and 3.78 (sd = 0.88), respectively. Finally, the mean value of AP is 4.35 (sd = 0.75). High reliability also characterizes all of the variables (acceptable level a > 0.7; Kerlinger & Lee, 2000).

### 5.1. Direct Effects

As shown in Table 2, transformational leadership (b = 0.49, *p* < 0.01), inclusive leadership (b = 0.31, *p* < 0.05), as well as adaptive leadership (b = 1.06, *p* < 0.01) are positively related to employees’ adaptive performance. Thus, hypotheses 1, 2, and 3 are confirmed. Further, both promotive voice behavior (b = 0.96, *p* < 0.01), and prohibitive voice behavior (b = 1.45, *p* < 0.01) are positively related to employees’ adaptive performance. Thus, hypotheses 4 and 5 are also confirmed.

### 5.2. Mediation Effects

The research model was examined using the structural equation modeling technique (hereafter, SEM) with maximum likelihood estimation using the analysis of moment structures program (AMOS version 24). First, we developed and tested the measurement model via confirmatory factor analysis (hereafter, CFA), and second, the SEM technique was used to examine all path coefficients. We employed several frequently accepted model fit adequacy indexes such as normed chi-square (χ^2^/df), standardized root mean square residual (SRMR), goodness offit index (GFI), comparative fit index (CFI), and root mean square error of approximation (RMSEA). The model fit indexes, which are presented in Table 3 together with the acceptable cutoff points, indicate a good fit for our model.

**Figure 2:**
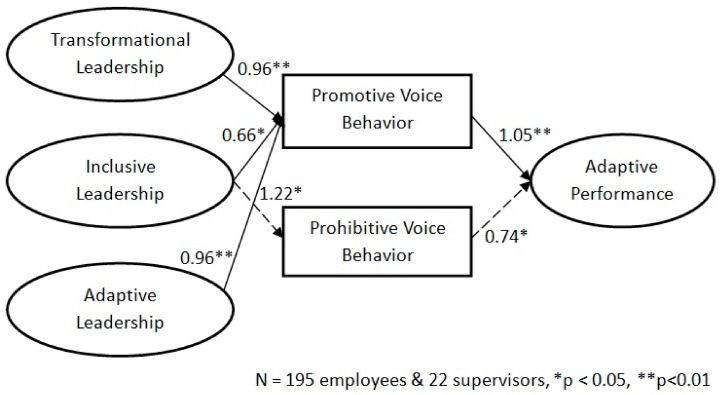
Structural equation modeling results for the hypothesized model (Note: * *p* < 0.05, ** *p* < 0.01; created by the author).

Subsequently, we conducted a power analysis by using the program by MacCallum et al. (1996), which indicated power levels above 0.95. This finding indicates that the sample size was suitable for minimizing type II errors. Further, specific path coefficients were tested to examine whether the relationships of the proposed model were confirmed by the empirical evidence. As shown in Figure 2, promotive voice behavior mediates the relationships among transformational, inclusive, and adaptive leadership and employees’ adaptive performance (b = 1.05, *p* < 0.01), consistent with hypothesis 6. Prohibitive voice behavior also mediates the relationship between inclusive leadership and employees’ adaptive performance (b = 0.74, *p* < 0.05). Thus, hypothesis 7 is merely supported.

## 6. Discussion

Similar to social exchange theory and the norm of reciprocity, the research findings suggest that there is a positive relationship between positive leadership approaches (i.e., transformational, inclusive, and adaptive) and employee adaptive performance. Quite similarly, Kaltiainen and Hakanen (2022) found that servant leadership practices may increase employees’ task and adaptive performance through the promotion of work engagement. Further, Katsaros (2022) showed that workplace belongingness mediates the relationship between inclusive leadership and employees’ change participation. Additionally, there is evidence that employee voice behavior (promotive and prohibitive) mediates the positive relationship between leadership and employee adaptive performance. Similarly, several scholars have observed that an anthropocentric leadership approach can increase employee voice behavior (e.g., Chen & Hou, 2016; Weiss et al., 2017; Zhang et al., 2015) and foster positive change-related behaviors (e.g., Maynes et al., 2024; Naqvi, 2020; Potnuru et al., 2023).

### 6.1. Theoretical Implications

This study yields several significant theoretical implications. The research findings highlight the significance of employee voice behavior (both promotive and prohibitive) during times of change, an aspect that may have been overlooked in previous studies. The present research suggests that employee voice behavior acts as an important personal resource, which is positively affected by leadership and, in turn, may increase employees’ adaptive performance. This finding also adds to change management literature’s pursuit of means to accomplish better employee adaptation in times of change (Vakola et al., 2021). In addition, the findings have important implications for sociological and psychological theories, including social exchange theory and the norm of reciprocity, as they demonstrate that employee voice behavior (promotive and prohibitive) is a key behavioral motivator. Furthermore, these findings broaden our understanding of these motivators in the context of change. Finally, the research enhances the theory of change in tourism (Twining-Ward et al., 2018) by highlighting the significance of employee voice behavior (both promotive and prohibitive) within the tourism sector.

### 6.2. Practical Implications

From a practical standpoint, this study offers four important implications for leaders and managers. First, it highlights that employee voice behavior (both promotive and prohibitive) mediates the relationship between leadership and employee adaptive performance. Therefore, leaders should concentrate on enhancing their Gen Z employees’ voice behavior—both promotive (suggestions and ideas) and prohibitive (raising concerns or objections)—during organizational change. For instance, they can cultivate a culture of openness by creating safe channels for employees to express their thoughts without fear of retaliation, conducting regular check-ins, establishing opendoor policies, and modeling transparency (Crone, 2020). Additionally, providing training and resources, such as voice training programs and conflict resolution skills, is essential. Recognizing and rewarding contributions through acknowledgment and incentives is also important (Naqvi, 2020). Implementing feedback mechanisms, like regular surveys and anonymous channels for feedback, can further support this effort (Crone, 2020). Further, empowering employees by involving them in decisionmaking and encouraging their autonomy is crucial, as is fostering trust and psychological safety through strong relationships and a supportive environment (Naqvi, 2020). By adopting these strategies, organizations can effectively promote both promotive and prohibitive employee voice behavior, resulting in a more engaged and adaptive workforce (Zhang et al., 2024). Second, the research findings indicate that a positive leadership approach (i.e., transformational, inclusive, and adaptive) is positively associated with employee adaptive performance. Moreover, Gen Z employees are particularly inclusive, value personal contributions, seek meaningful changes, and demand that companies adopt more anthropocentric, diverse, and inclusive practices and strategies (Kratz, 2023). Therefore, tourism leaders and change management practitioners should implement adaptive, inclusive, and participatory practices during organizational change. This includes fostering psychological safety, encouraging group involvement, ensuring employees feel respected and valued, allowing for influence in decisionmaking, promoting authenticity, and recognizing and advancing diversity (Shore et al., 2018). Third, organizations can greatly benefit from training their leaders to become supporters and enablers of employee voice behavior. This may involve various strategies (Scudder, 2020), such as mindset training (e.g., distinguishing between growth and fixed mindsets), developing empathy and listening skills (e.g., enhancing leaders’ emotional intelligence to better understand and respond to employees’ needs), creating safe spaces (e.g., environments where employees feel comfortable sharing ideas, providing feedback, and taking risks), celebrating ideas (e.g., recognizing both successful and unsuccessful creative efforts to reinforce the value of all contributions), and implementing iterative training (e.g., incorporating feedback and best practices to maintain relevance and effectiveness). Four, there is evidence that positive psychology may improve employee adaptive performance (Tang et al., 2024). Thus, managers may considerably benefit if they use the well-being theory proposed by the PERMA model (Seligman, 2018) and try to positively influence their employees’ positive emotions, engagement, relationships, meaning, and accomplishments in the workplace. Neglecting these practices could lead to ineffective human resource and change management strategies.

Like any research, this study has limitations. Methodologically, the results may be influenced by specific temporal or contextual factors. Since the study was conducted during a significant cultural shift, further research is needed to verify whether the relationships identified hold true in different organizational settings. Additionally, participants may have been influenced by social desirability bias (Podsakoff et al., 2003), which could lead them to respond in ways that are viewed positively by others, potentially obscuring their true feelings. Moreover, employee adaptive performance is undoubtedly a multidimensional construct and, as such, this study does not fully encompass its potential complexity. Therefore, further research that builds upon these findings by using larger and more representative samples would be highly valuable. Additionally, more investigation is required to explore the relationship between leadership and employee adaptive performance. For example, researchers could investigate the role of additional contextual factors (e.g., involvement, participation, role clarity, manager support, and constructive change-related conflicts) and personal resources (e.g., flexibility, resilience, perseverance, and enthusiasm). These insights would be beneficial for both the theory and practice of change management. Finally, we encourage the use of alternative theoretical frameworks to explore the full range of interactions influencing employee voice behavior (both promotive and prohibitive), thereby fostering further developments in this research area.

## Figures and Tables

**Table 1:** Means, standard deviations, reliabilities, and correlations.

Variables		Mean	SD	Alpha	1	2	3	4	5	6
1.	Gender ^a^	-	-	-						
2.	Transformational leadership	4.15	1.08	0.80	0.09					
3.	Inclusive leadership	4.22	1.11	0.82	−0.12 *	0.52				
4.	Adaptive leadership	3.88	0.77	0.86	0.24 *	0.36	0.44			
5.	Promotive voice behavior	4.44	1.01	0.84	0.11 *	0.51 *	0.32 *	0.22 *		
6.	Prohibitive voice behavior	3.78	0.88	0.81	−0.06 *	0.36	−0.26	0.21 *	0.42	
7.	Adaptive performance	4.35	0.75	0.79	0.04 *	0.22 **	0.54 *	0.90 *	0.96 **	1.45 **

N = 195 employees and 22 supervisors, * *p* < 0.05, ** *p* < 0.01, ^a^ Men = 0; Women = 1.

**Table 2:** Regression analysis results—testing of hypotheses 1–5.

Variables	H1	H2	H3	H4	H5
(Constant)	2.71 **	7.42 **	4.22 **	3.78 **	5.19 **
Gender	0.22 **	0.25 **	0.08 **	0.54 **	0.73 **
Transformational leadership	0.49 **				
Inclusive leadership		0.31 **			
Adaptive leadership			1.06 **		
Promotive voice behavior				0.96 **	
Prohibitive voice behavior					1.45 **
R^2^	0.44 **	0.28 **	0.46 **	0.55 **	0.33 **
F	6.22 **	4.22 *	3.78 **	7.25 *	6.44 *

N = 195 employees and 22 supervisors, * *p* < 0.05, ** *p* < 0.01.

**Table 3:** Model fit indexes.

Model Fit	Mediated Model	Cutoff Point	Reference
Normed Chi-Square (χ^2^/df)	2.10	<3	Qing et al. (2019)
Standardized Root Mean Square Residual (SRMR)	0.03	<0.05	Iacobucci (2010)
Goodness Fit Index (GFI)	0.96	>0.95	Hair (2011)
Comparative Fit Index (CFI)	0.97	>0.95	Hair (2011)
Rootmean-Square Error of Approximation (RMSEA)	0.05	<0.06	Iacobucci (2010)

## Data Availability

Data are unavailable due to privacy or ethical restrictions.
